# Prolactin does not seem to mediate the improvement on insulin resistance markers and blood glucose levels related to breastfeeding

**DOI:** 10.3389/fendo.2023.1219119

**Published:** 2023-08-30

**Authors:** Julia Martins de Oliveira, Patricia Medici Dualib, Alexandre Archanjo Ferraro, Camila Rodrigues de Souza Carvalho, Rosiane Mattar, Sérgio Atala Dib, Bianca de Almeida-Pititto

**Affiliations:** ^1^ Post-Graduation Program in Endocrinology and Metabology, Universidade Federal de Sao Paulo, São Paulo, Brazil; ^2^ Department of Medicine, Universidade Federal de Sao Paulo, São Paulo, Brazil; ^3^ Department of Pediatrics, Universidade de São Paulo, São Paulo, Brazil; ^4^ Department of Obstetrics, Universidade Federal de Sao Paulo, São Paulo, Brazil; ^5^ Department of Preventive Medicine, Universidade Federal de Sao Paulo, São Paulo, Brazil

**Keywords:** breastfeeding, gestational diabetes mellitus, prolactin levels, insulin resistance, glucose tolerance, postpartum

## Abstract

**Introduction:**

The prevalence of type 2 diabetes mellitus (T2DM) is increasing worldwide. Strategies to decrease this risk should be strongly encouraged. Lactation has been associated, for the mother, with reduction in future T2DM risk in several studies. The mechanisms behind this phenomenon, however, are poorly understood. The aims of this study were, first, to compare blood glucose levels and markers of insulin resistance (MIR) in early postpartum women with overweight/obesity according to their breastfeeding status and, second, to evaluate whether prolactin (PRL) levels could mediate improvements in these parameters.

**Methods:**

The prospective study followed 95 women older than 18 years from early pregnancy for up to 60 to 180 days postpartum. All participants had a BMI > 25 kg/m^2^ and a singleton pregnancy. At each visit, questionnaires and clinical and biochemical evaluations were performed. Participants were divided into two groups according to the breastfeeding status as “yes” for exclusive or predominant breastfeeding, and “no” for not breastfeeding.

**Results:**

Breastfeeding women (*n* = 44) had significantly higher PRL levels [47.8 (29.6–88.2) vs. 20.0 (12.0–33.8), *p*< 0.001]. They also had significantly lower fasting blood glucose levels [89.0 (8.0) vs. 93.9 (12.6) mg/dl, *p* = 0.04], triglycerides (TG) [92.2 (37.9) vs. 122.4 (64.4) mg/dl, *p* = 0.01], TG/HDL ratio [1.8 (0.8) vs. 2.4 (1.6) mg/dl, *p* = 0.02], TyG index [8.24 (0.4) vs. 8.52 (0.53), *p* = 0.005], fasting serum insulin [8.9 (6.3–11.6) vs. 11.4 (7.7–17.0), *p* = 0.048], and HOMA-IR [2.0 (1.3–2.7) vs. 2.6 (1.6–3.9), *p* = 0.025] in the postpartum period compared to the non-breastfeeding group. Groups were homogeneous in relation to prevalence of GDM, pre-gestational BMI, as well as daily caloric intake, physical activity, and weight loss at postpartum. Linear regression analysis with adjustments for confounders showed a statistically significant association of breastfeeding with fasting blood glucose [−6.37 (−10.91 to −1.83), *p* = 0.006], HOMA-IR [−0.27 (−0.51 to −0.04), *p* = 0.024], TyG index [−0.04 (−0.06 to −0.02), *p* = 0.001], and TG/HDL ratio [−0.25 (−0.48 to −0.01), *p* = 0.038]. Mediation analysis showed that PRL did not mediate these effects. Sensitivity analyses considering different cutoffs for PRL levels also did not show modification effect in the mediation analyses.

**Conclusion:**

Breastfeeding was associated with improvement in glucose metabolism and MIR 60 to 180 days after birth in overweight and obese women, even when adjusted for confounders. PRL levels were not found to mediate the association between breastfeeding and improvement in MIR.

## 1 Introduction

Type 2 diabetes mellitus (T2DM) is a medical condition with increasing prevalence worldwide. Data from the IDF Diabetes Atlas from 2021 estimate that 537 million individuals are affected by this condition, and what is even more alarming, a 46% increase in prevalence is expected by 2045, mainly in low- and middle-income countries (1, 2). Unhealthy lifestyle, weight gain, and physical inactivity are some of the modifiable factors that explain the increase in T2DM prevalence. Gestational diabetes mellitus (GDM) is also a well-known risk factor and a predictor of T2DM after pregnancy: studies have shown that up to 50% of women with GDM develop T2DM at some time in life (3–5). It is important to note that the global prevalence of GDM was reported to be 14.7% in a recent meta-analysis from 2021 (6). In this sense, lifestyle interventions that reduce the onset of T2DM, mainly among individuals at higher risk, should be strongly encouraged. An intervention that has been associated with risk reduction of future diabetes is lactation, which might also be relevant in the context of the high prevalence of GDM and T2DM (7, 8).

Breastfeeding has been associated with several favorable metabolic effects for mothers and their offspring and its effects last for years after weaning (4, 7, 9, 10). Chouinard-Castonguay et al., for example, demonstrated in 144 patients 4 years after delivery that women who had breastfed for more than 10 months had significantly lower levels of fasting insulin and improved insulin secretion index when compared to those who had breastfed for less than 10 months (7). The prospective study CARDIA followed 704 women for 20 years and observed an inverse relation between duration of lactation and risk of developing metabolic syndrome (MS). Interestingly, a greater risk reduction of MS was observed among patients with a history of GDM (10). The SWIFT study followed 1,010 women with recent GDM and without T2DM [confirmed by the oral glucose tolerance test (OGTT)] for a median time of 1.8 years after birth and identified that a longer duration and intensity of lactation was associated with lower incidence of T2DM in that period, even when adjusted for age, maternal, and perinatal risk factors (11).

These studies corroborate the potential benefits of lactation for improving metabolic parameters and for reducing future risk of T2DM, even among women with a history of GDM. The mechanisms by which these improvements occur are, however, poorly understood (12, 13). In this context, the hypothesis that biomarkers produced during lactation, such as prolactin (PRL), could improve markers of insulin resistance (MIR) has emerged.

Pregnancy and breastfeeding increase the production and secretion of PRL by the adenohypophysis (14). *In vitro* studies have shown that the pancreatic β cells have PRL receptors that, when activated, stimulate cell proliferation and insulin secretion (15). This mechanism is especially important as an adaptative response to the increased insulin resistance that occurs during pregnancy. Some studies have also suggested that increased PRL levels in the postpartum period are related to improvement in MIR. A study conducted by Ozisik et al., for instance, found that, in 33 postpartum women, PRL levels were inversely correlated to HbA1c and 2-h serum C-peptide (16). The SWIFT study evaluated the relationship between PRL and T2DM development in women with recent GDM and found that lower levels of PRL were associated with increased future risk of T2DM and that in normoglycemic women higher PRL was associated with improved insulin sensitivity (4).

Considering these aspects, our goal was, first, to compare metabolic parameters in early postpartum according to breastfeeding status and, second, to evaluate the potential mediation effect of PRL on these parameters in women with overweight/obesity and GDM.

## 2 Methods

### 2.1 Study design and subjects

The study had a prospective design and enrolled a convenience sample between September 2018 and December 2019. All pregnant women with a BMI > 25 kg/m^2^ and with a singleton pregnancy attending the General Gestation Out-patient Clinic of Obstetrics Division and the Gestational Diabetes Out-patient Clinic of the Diabetes Center of the Federal University of São Paulo, SP, Brazil, were invited to participate. Only overweight and obese patients were included in this study so that the benefits of breastfeeding could be studied in women at higher risk of developing glucose intolerance. Exclusion criteria included the following: known auto-immune disease, chronic diseases, and use of any medication (mainly metformin), except for those regularly prescribed during pregnancy. Initially, 143 women were included. Of these, 74 were normo-glycemic and 69 had a diagnosis of GDM. In the postpartum period (60 to 180 days after delivery), 95 women with their respective offspring were evaluated. Of these, 44 women were breastfeeding and 45 had had a diagnosis of GDM during pregnancy.

For the diagnosis of GDM, the IAPDSG criteria were used, which are similar to the Brazilian guideline for GDM from the Brazilian Society of Diabetes (17, 18). However, in our study, GDM was diagnosed either in the first trimester with a fasting plasma glucose greater than 100 mg/dl or in the third trimester with at least two altered points in the 75-g OGTT (>92, >180, and >153 mg/dl at 0, 60, and 120 min, respectively). These criteria were used to exclude borderline cases of GDM so that we could better compare insulin-resistant and normoglycemic controls.

During pregnancy, glucose levels were thoroughly followed by the “Diabetes and gestation” Team (composed of endocrinologists, nurses, obstetrics, and nutritionists). Insulin was prescribed when necessary to achieve adequate glycemic control: a fasting capillary glucose of up to 95 mg/dl and a 1-h post-prandial of up to 140 mg/dl. Capillary glucose was the main parameter for optimal control during pregnancy since glycated hemoglobin (HbA1c) was not assessed in all participants at that time.

The institutional ethics committee of the Federal University of São Paulo approved the study (Protocol Number: CAAE: 89108618.0.0000.5505). All participants signed an informed consent.

### 2.2 Standardized questionnaires

Participants were followed up during pregnancy and at postpartum (at 60 to 180 days after delivery). They answered structured questionnaires regarding four different moments: immediately before diagnosis of pregnancy, during the 1st/2nd trimesters, during the 3rd trimester, and, finally, 60 to 180 days postpartum.

The information was obtained with the use of standardized questionnaires applied by trained interviewers. Collected data included the following: socio-demographic information, life habits, personal and familial past medical history, diet, pre-gestational BMI, weight gain during pregnancy, parity, gestational week of delivery, mode of delivery, maternal–fetal complications, use of medications during pregnancy, consumption of alcohol and tobacco, and breastfeeding.

High education level was defined by at least 14 years of schooling. Individuals were physically active if they performed at least 150 min of at least moderate-intensity physical activity per week.

### 2.3 Anthropometry and blood pressure

Weight was obtained on a digital scale (Rice Lake, São Paulo) with a 100-g precision and a height accuracy of 0.5 cm. These measurements were used to calculate the Body Mass Index (BMI). The neck circumference was measured immediately below the cricoid cartilage and perpendicular to the neck’s long axis with the use of a non-flexible tape (cm) and with the participants seated. The waist circumference was measured between the iliac crest and the last ribs with a flexible tape (cm). Blood pressure was obtained three times after a 5-min rest in the sitting position, using a mercury sphygmomanometer adjusted to the brachial circumference. The final values of systolic and diastolic pressure represent the arithmetic mean of the last two measurements.

### 2.4 Dietary assessment

All foods and beverages consumed over 3 days, including those consumed outside participants’ homes, were registered in a standardized form. To estimate the size of the portions with more accuracy, the nutritionist demonstrated how to record the information using traditional homemade utensils (cups, cutlery, and plates) and food models. Registration was made on alternate days and necessarily covered a weekend day (19). The total energy value of macro- and micronutrients was calculated using the Diet Pro software, using as reference the Brazilian Food Composition Table (TBCA) (20).

### 2.5 Laboratory tests

In the postpartum period (60 to 180 days after delivery) participants were subject to laboratory and clinical evaluation in a previous scheduled data. Blood samples were collected after an overnight fast, and an OGTT was subsequently performed. The samples were immediately centrifuged and analyzed by a private certificate laboratory. Plasma glucose was determined by the glucose oxidase method. The concentrations of total cholesterol, HDL-c, and triglycerides were measured by enzymatic colorimetric methods, processed in an automatic analyzer. LDL-c and VLDL-c concentrations were obtained by difference, using the Friedewald equation. Insulin was measured by the chemiluminescence method.

Insulin resistance was evaluated by the HOMA-IR (homeostasis model assessment—insulin resistance), the Triglycerides–glucose index (TyG index), and the Triglyceride-to-HDL-cholesterol ratio (TG/HDL). The following equations were used:


$$\text{HOMA-IR}=\frac{\left[\text{Fasting Insulin }\left(\text{µ}\frac{\text{mU}}{\text{L}}\right)\text{x Fasting Glucose }\left(\frac{\text{mmol}}{\text{L}}\right)\right]}{22,5}$$

(21)


$$\text{TyG index}=\frac{\text{ln }[\left(\text{Fasting triglycerides }\left(\text{mg}/\text{dl}\right)\;\times \text{ Fasting Glucose }\left(\text{mg}/\text{dl}\right)\right]}{2}$$

(22)

Insulin secretion was estimated through HOMA-β (Homeostasis model assessment of β function), calculated using the equation:


$$\text{HOMA-β}=\frac{\left[20\text{ x Fasting Insulin }\left(\frac{\text{μUI}}{\text{mL}}\right)\right]}{\left[\text{Fasting Glucose }\left(\frac{\text{mmol}}{\text{L}}\right)–\;3,5\right]}$$

(19)

### 2.6 Definitions for analysis

Breastfeeding (exposure variable) was evaluated on the postpartum visit. Emphasis was given on how long the mother had been breastfeeding, if she was still breastfeeding on the day of the visit, and if other types of liquids or foods (water, tea, and fruits) were given. We considered breastfeeding as predominant or exclusive if the main nutritional source for the baby was breastmilk, even if water and/or tea and/or fruits were concomitantly offered (without use of formulas or cow milk).

The outcome variables were those that represent glucose metabolism and insulin resistance, such as fasting and 2-h plasma glucose after an OGTT, and MIR, such as HOMA-IR, TG/HDL-cholesterol ratio, TG, and glucose (Tyg) index and HOMA-β.

### 2.7 Statistical analysis

Variables with a non-normal distribution were logarithmized so that a normal distribution was obtained. Continuous variables with a normal distribution were expressed as mean and standard deviation (SD), while non-normally distributed variables were expressed as median and interquartile range (IQR) and were log-transformed for the statistical analysis. Categorical variables were expressed as frequency and percentage (%).

Clinical and laboratory variables and maternal–fetal outcomes were compared by Student’s *t*-test or Mann–Whitney test (continuous variables), as appropriate, or Chi-squared test (categorical variables) according to the status of breastfeeding.

**Figure 1:**
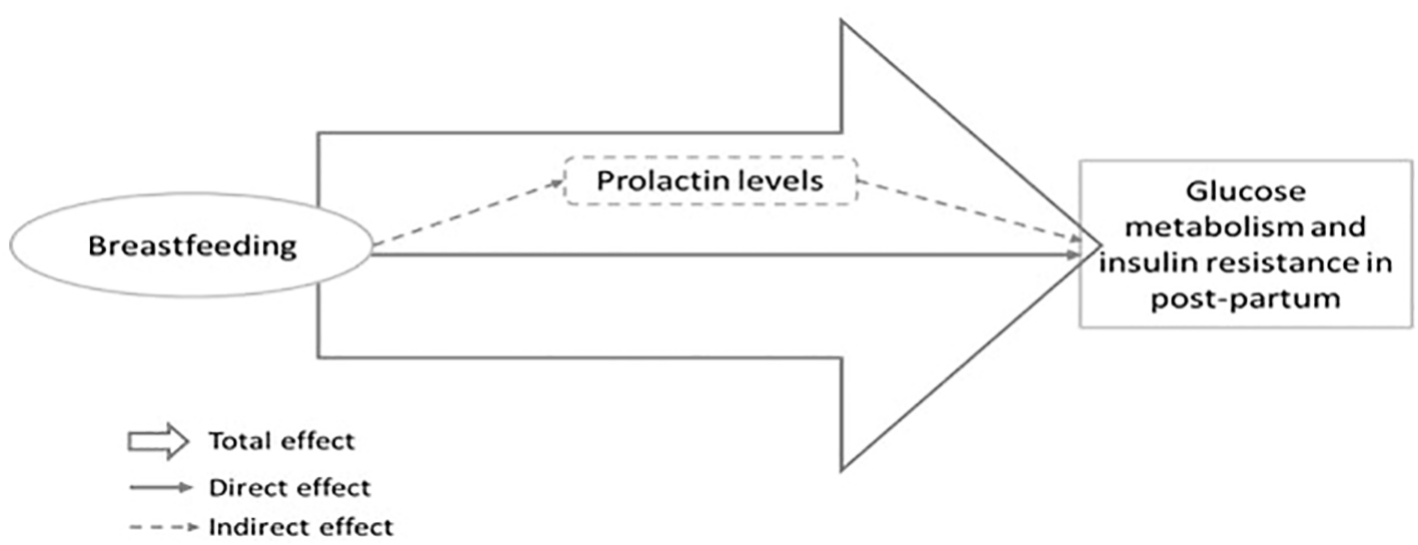
Theoretical model for the investigation of the total, direct and indirect effect (mediated by prolactin levels) of breastfeeding on glucose metabolism and insulin resistance in post-partum.

Associations between exposure (breastfeeding) and outcome variables (glucose and insulin levels and MIR: HOMA-IR, TyG index, TG/HDL-c, and HOMA-β) were initially analyzed by linear regression, adjusted for potential confounders. Correlation analyses were performed between PRL levels and outcome variables by Pearson correlation coefficients. Mediation analyses were performed to evaluate the total, direct, and indirect effect of breastfeeding on MIR variables, considering PRL levels as a mediator (**Figure 1**), using the command *medeff [(regress mediator exposure adjustments for confounders) (regress outcome mediator exposure adjustments for confounders), treat (exposure) med(mediator)*] from the statistical software STATA.

To identify possible confounders in these associations, Directed Acyclic Graphs (DAGs) were used. A DAG is a causal diagram based on a theoretical mathematical model that is used to visually represent the relationships between variables (23, 24). Its final objective is to identify the minimum sufficient adjustment variables to prevent biases and overadjustments. For mediation analysis, DAG was also performed to evaluate potential confounders between breastfeeding and PRL levels and also between PRL levels and glucose levels (Glu) and MIR. The DAG that associates breastfeeding with Glu and MIR is shown in the **Supplementary Material (S1)**. In this theoretical model, the minimal sufficient adjustment set for estimating the total effect of breastfeeding on glucose tolerance were neonatal birth weight, GDM, parity, scholarity, pre-gestational BMI, type of delivery, and weight gain during pregnancy. This model was created by DAGitty software, version 3.0 (www.dagitty.net) (25).

For statistical analysis, the software *Statistical Package for the Social Sciences* ^®^, version 16.0 (SPSS Incorporation, 2000) was used. The mediation analysis was performed using the statistical software STATA^®^. *p* was considered statistically significant if<0.05.

## 3 Results

Ninety-five women completed the study, 44 of whom were breastfeeding at the time of the postpartum visit. Of these 44 participants, 11 were also offering their babies water and/or tea and/or fruits, but breastmilk was the predominant source of calories. **Table 1** compares participants that were breastfeeding with those who were not between 60 and 180 days after delivery. Initial socio-demographic characteristics, life habits, and personal and familial past medical history were similar between groups, including pre-gestational BMI. A total of 45 participants had had GDM (20 from the non-breastfeeding group and 25 from the breastfeeding group) and 50 had not. The prevalence of GDM did not differ among breastfeeding groups. In the postpartum period, 2 patients were diagnosed with T2DM (both were in the non-breastfeeding group) and 21 patients were diagnosed with pre-diabetes (10 in the non-breastfeeding and 11 in the breastfeeding group).

**Table 1:** Participants’ characteristics in the pre-gestational, third trimester and in the postpartum periods, according to breastfeeding status.

Pre-gestational period	Breastfeeding		*p*
	No (*n* = 51)	Yes (*n* = 44)	
Age (years)	30.3 (7.3)	31.1 (5.8)	0.560
Race, white, *n* (%)	22 (43.1)	22 (50.0)	0.717
High educational level, *n* (%)	12 (23.5)	14 (31.8)	0.366
Three or more previous gestations, *n* (%)	18 (35.3)	17 (38.6)	0.736
Family history of diabetes mellitus, *n* (%)	18 (35.3)	15 (34.1)	0.902
Physical activity before pregnancy, *n* (%)	23 (45.1)	17 (38.6)	0.525
Pre-gestational weight (kg)	76.3 (12.0)	78.3 (11.4)	0.418
Pre-gestational BMI (kg/m^2^)	29.3 (4.0)	30.4 (3.9)	0.176
BMI ≥ 30 kg/m^2^, *n* (%)	17 (33.3)	20 (45.5)	0.227
3rd trimester			
Physical activity, *n* (%)	2 (4.4)	2 (5.3)	0.862
Smoking, *n* (%)	0	0	–
Alcohol consumption, *n* (%)	0	0	–
Insulin use during pregnancy, *n* (%)	12 (24.0)	14 (32.6)	0.359
Hypertension during pregnancy, *n* (%)	7 (13.7)	6 (13.6)	0.990
GDM, *n* (%)	20 (39.2)	25 (56.8)	0,087
Pre-eclampsia, *n* (%)	3 (5.9)	1 (2.3)	0.621
Postpartum period			
Gestational age at birth (weeks)	38.9 (1.2)	38.5 (1.2)	0.128
Vaginal birth, *n* (%)	27 (52.9)	26 (59.1)	0.547
Birth weight (kg)	3.4 (0.4)	3.3 (0.5)	0.303
Weight gain during pregnancy (kg)	11.6 (5.5)	8.1 (6.2)	0.006
Physical activity at postpartum, *n* (%)	2 (3.9)	2 (4.5)	0.880
Diet—daily calorie consumption (kcal)^*^	1,743.3 (1,604.8–2,382.1)	1,799.8 (1,421.5–2,501.4)	0.737
BMI (kg/m^2^)	29.8 (4.4)	29.5 (3.6)	0.723
BMI ≥ 30 kg/m^2^, *n* (%)	17 (33.3)	15 (34.1)	0.938
Waist circumference (cm)	93.4 (9.9)	93.5 (7.8)	0.940
Neck circumference (cm)	35.3 (2.3)	34.9 (3.1)	0.519
Weight variation at postpartum (kg)	−9.3 (5.4)	−9.5 (4.1)	0.811
Systolic blood pressure (mmHg)	113.4 (10.3)	115.8 (9.0)	0.237
Diastolic blood pressure (mmHg)	73.6 (7.7)	73.7 (6.5)	0.967
Total cholesterol (mg/dl)	193.1 (45.9)	193.1 (40.4)	0.997
HDL-cholesterol (mg/dl)	55.2 (11.3)	55.0 (11.1)	0.925
LDL-cholesterol (mg/dl)	113.6 (40.2)	117.8 (35.3)	0.593
Triglycerides (mg/dl)^#^	122.4 (70.0–164.0)	92.2 (62.2–120.8)	0.013
Fasting blood glucose (mg/dl)	93.9 (12.6)	89.0 (8.0)	0.036
2-hour post OGTT blood glucose (mg/dl)	112.8 (37.8)	103.5 (27.3)	0.185
HbA1c (%)	5.5 (0.4)	5.5 (0.3)	0.880
Fasting serum insulin (µU/ml) ^#^	12.5 (7.7–17.0)	10.0 (6.3–11.6)	0.048
HOMA-IR^#^	2.6 (1.6–3.9)	2.0 (1.3–2.7)	0.025
HOMA-β^#^	125.8 (95.8–174.5)	121.4 (99.6–188.4)	0.675
TG/HDL ratio^#^	2.4 (1.3–3.3)	1.8 (1.1–2.2)	0.038
TyG index^#^	8.5 (8.1–8.9)	8.2 (8.0–8.6)	0.006
Prolactin (ng/dl) ^#^	20.0 (12.0–33.8)	47.8 (29.6–88.2)	<0.001

Data are expressed as mean (standard deviation) or as median (interquartile interval)^#^ when distribution is non-normal. Categorical variables are presented as n (percentile). ^#^Non-normal variables were log-transformed for statistical analysis. Student’s t-test was used for continuous variables. Chi-square test was used for categorical variables. Physical activity: >150 min of exercise per week; High educational level, at least 14 years of schooling; >BMI, body mass index; TG, triglycerides; HDL, HDL-cholesterol; HbA1c, glycated hemoglobin; GDM, gestational diabetes mellitus; OGTT: oral glucose tolerance test; HOMA-IR (Homeostatic Model Assessment for Insulin Resistance) was calculated using the formula: [fasting insulin (µmU/L) × fasting glucose (mmol/L)]/22.5; HOMA-β (Homeostasis model assessment of β function) was calculated using the formula: fasting insulin (µU/ml) × 20/(fasting glucose (mg/dl) × 0.0555) − 3.5; triglyceride and glucose (Tyg) index was calculated using the formula: ln[(TG mg/dl × glucose mg/dl)/2].

Regarding the postpartum data (**Table 1**), we observed that BMI was also similar between groups, but the group that was not breastfeeding had a significantly greater weight gain during pregnancy [11.6 (5.5) vs. 8.1 (6.2) kg, *p* = 0.006]. Other anthropometric data (neck circumference and waist circumference) were similar between groups, as well as the frequency of women who were physically active and the total daily caloric intake in postpartum period. Women who were breastfeeding had significantly higher PRL levels [47.8 (29.6–88.2) vs. 20.0 (12.0–33.8) ng/dl, *p*< 0.001], lower TG [92.2 (62.2–120.8) vs. 122.4 (70.0–164.0) mg/dl, *p* = 0.006], lower fasting blood glucose [89.0 (8.0) vs. 93.9 (12.6) mg/dl, *p* = 0.036], and lower fasting insulin levels [10 (6.3–11.6) vs. 12.5 (7.7–17.0) µU/ml, *p* = 0.048] than women who were not breastfeeding.

**Figure 2:**
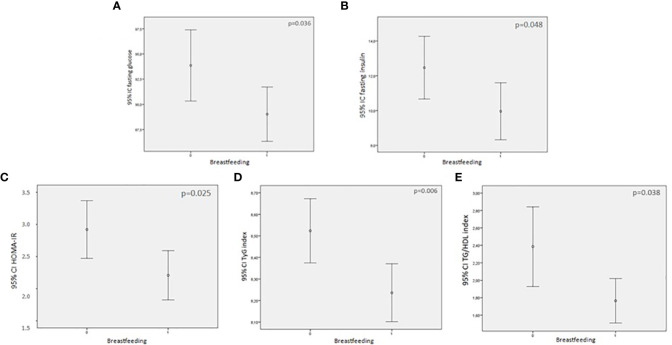
Comparison between glucose levels and markers of insulin resistance (MIR) between breastfeeding and non=breastfeeding women (0 = not breastfeeding; 1= breastfeeding. **(A)** fasting blood glucose and breastfeeding; **(B)** fasting serum insulin and breastfeeding; **(C)** HOMA-IR and breastfeeding; **(D)** TyG index and breastfeeding; **(E)** TG/HDL index and breastfeeding.

The MIR were significantly lower in the group that was breastfeeding as shown in **Figure 2**: TG/HDL ratio [1.8 (1.1–2.2) vs. 2.4 (1.3–3.3) *p* = 0.020], TyG index [8.2 (8.0–8.6) vs. 8.5 (8.1–8.9), *p* = 0.006], and HOMA-IR [2.0 (1.3–2.7) vs. 2.6 (1.6–3.9), *p* = 0.025].

Linear regression analysis was performed to evaluate the association of breastfeeding with MIR and glucose levels (**Table 2**). The Crude model (without adjustments) shows that fasting blood glucose, fasting serum insulin, HOMA-IR, TyG index, and TG/HDL ratio were inversely associated with breastfeeding. When we adjusted the model for the parameters indicated by the DAG model (parity, scholarity, pre-gestational BMI, GDM, type of delivery, weight gain during pregnancy, and neonatal birth weight), a statistically significant and inverse association was still observed between breastfeeding and fasting serum glucose [−6.37 (−10.91 to −1.83), *p* = 0.006], HOMA-IR [−0.27 (−0.51 to −0.04), *p* = 0.024], TyG index [−0.04 (−0.06 to −0.02), *p* = 0.001], and TG/HDL ratio [−0.25 (−0.48 to −0.01), *p* = 0.038]. We also analyzed the association between breastfeeding and HOMA-β, but no significant correlation was found (data not shown).

**Table 2:** Linear regression analysis considering MIR variables as dependent variables and breastfeeding as the main independent variable of interest.

	Crude			Model 1		
	B	95% CI	*p*	β	95% CI	*p*
**Fasting blood glucose**	−4.84	−9.35 to −0.33	0.036	−6.37	−10.91 to −1.83	0.006
**Fasting serum Insulin** ^#^	−0.22	−0.44 to −0.00	0.048	−0.21	−0.43 to 0.13	0.065
**HOMA-IR** ^#^	−0.27	−0.50 to −0.03	0.025	−0.27	−0.51 to −0.04	0.024
**TyG index** ^#^	−0.03	−0.06 to −0.01	0.006	−0.04	−0.06 to −0.02	0.001
**TG/HDL ratio** ^#^	−0.24	−0.46 to −0.01	0.038	−0.25	−0.48 to −0.01	0.038

Model 1: adjusted for parity, scholarity, pre-gestational BMI, GDM, type of delivery, weight gain during pregnancy and neonatal birth weight.

Fasting glucose is expressed in mg/dL.HOMA-IR (Homeostatic Model Assessment for Insulin Resistance) was calculated using the formula: [fasting insulin (µmU/L) × fasting glucose (mmol/L)]/22.5; triglyceride and glucose (Tyg) index was calculated using the formula: ln[(TG mg/dl × glucose mg/dl)/2]. ^#^Log-transformed values of outcomes for analyses.

PRL levels were inversely associated with glucose metabolism and MIR as presented in **Table S1** in the **Supplementary Material**. Statistically significant and inverse correlations between PRL levels and TyG index (−0.266, *p* = 0.028) and between PRL levels and TG/HDL ratio were observed (−0.243, *p* = 0.018), while the correlation with HOMA-IR was borderline (*p* = 0.091).

Finally, **Table 3** shows the mediation analysis performed to determine the total and direct effect of breastfeeding and the indirect effect of breastfeeding, via PRL, on MIR and glucose levels. Breastfeeding had a total effect on fasting blood glucose [−6.18 (−10.47 to −1.85)], fasting serum insulin [−0.27 (−0.50 to −0.02)], and TyG index [−0.04 (−0.06 to −0.01)]. When the indirect effect of PRL on these parameters was analyzed, we observed that this biomarker did not mediate the effects in fasting blood glucose, fasting serum insulin, and TyG index.

**Table 3:** Mediation analysis considering MIR (fasting blood glucose, fasting serum insulin, HOMA-IR, TyG index, and TG/HDL ratio) as dependent variables, breastfeeding as the interest independent variable, and prolactin as a potential mediator.

	Direct effect		Indirect effect		Total effect		% of total effect	
	β	95% CI	β	95% CI	β	95% CI	mediated	
**Fasting glucose**	−6.04	−11.32 to −0.92	−0.15	−2.56 to 2.51	−6.18	−10.47 to −1.85	0.02	0.01 to 0.08
**Fasting insulin^#^**	−0.20	−0.48 to 0.07	−0.06	−0.19 to 0.06	−0.27	−0.50 to −0.02	0.24	0.12 to 1.23
**HOMA-IR^#^**	−0.10	−0.39 to 0.19	−0.07	−0.20 to 0.07	−0.16	−0.41 to 0.09	0.33	−4.38 to 4.46
**TyG index^#^**	−0.03	−0.06 to −0.01	−0.01	−0.02 to 0.01	−0.04	−0.06 to −0.01	0.20	0.12 to 0.55
**TG/HDL ratio^#^**	−0.05	−0.34 to 0.24	−0.12	−0.26 to 0.03	−0.17	−0.42 to 0.10	0.58	−8.55 to 6.19

**^#^** Log-transformed values of outcomes for analyses. Fasting glucose in mg/dl.

HOMA-IR (Homeostatic Model Assessment for Insulin Resistance) was calculated using the formula: [fasting insulin (µmU/L) × fasting glucose (mmol/L)]/22.5; HOMA-β (Homeostasis model assessment of β function) was calculated using the formula: fasting insulin (μU/ml) × 20/(fasting glucose (mg/dl) × 0.0555) − 3.5; triglyceride and glucose (Tyg) index was calculated using the formula: ln[(TG mg/dl × glucose mg/dl)/2].

## 4 Discussion

In this study, women at high risk of developing glucose intolerance over time (overweight/obese and/or those with a past medical history of GDM) who were breastfeeding at 60 to 180 days postpartum had lower levels of fasting blood glucose, fasting serum insulin, and TG compared to women who were not breastfeeding in the same period. Besides that, all MIR were significantly lower in the breastfeeding group: TG/HDL ratio, TyG index, and HOMA-IR, even when adjusted by confounders identified through the DAG model. Higher PRL levels in breastfeeding women did not mediate the improvements in glucose levels or in MIR. It is important to mention that groups were homogeneous in most aspects, including potential confounding factors such as daily caloric intake, physical activity, and weight loss at postpartum. This homogeneity between groups strengthens the hypothesis that lactation *per se* plays a physiological role in improvement in MIR, as suggested by previous authors (11).

The HOMA-β index, a marker of insulin secretion, was similar among groups, suggesting that a short period of lactation, such as the one evaluated in this study, improves insulin resistance, but not insulin secretion. Perhaps this result would be different if a longer period of lactation was analyzed.

Many studies in literature have shown the multiple benefits of breastfeeding (4, 5, 7–11, 26–31). Our data are consistent with findings from previous studies that suggest that lactation is precociously associated with favorable metabolic changes. Lower glucose levels in the lactating mothers seen in our study have also been observed in other studies, and one of the mechanisms to explain this phenomenon is that the mothers’ blood glucose is used for milk production by the mammalian gland via insulin- and non-insulin-mediated pathways (32). It is known that lactation increases metabolic rates and mobilizes lipids into breast milk instead of the adipocytes (13). Women who are exclusively lactating require, on average, an additional 400 to 500 kcal daily (5, 12), which could facilitate weight loss and, hence, improve insulin resistance. In our study, however, weight loss was similar between groups; thus, this does not explain the difference seen in serum glucose levels and MIR.

In spite of the many beneficial aspects of lactation, only 43% of the participants were breastfeeding at the postpartum visit. This low prevalence is in accordance with other studies from the literature: a study from 2021 estimated that in low- to middle-income countries such as Brazil, the prevalence of exclusive breastfeeding under 6 months was 45.7% (33). Reasons for early weaning in our population included hypogalactia and difficulty to maintain lactation because of work.

The mechanisms through which breastfeeding that improve serum glucose and MIR are still poorly understood. One important hypothesis is the potential benefit in losing weight with breastfeeding; however, we reinforce that the weight change in postpartum period was similar in both groups. Even though weight gain during pregnancy was higher in women who did not breastfeed, we did not find differences in caloric intake between the groups when this was assessed at postpartum, and it is important to notice that the mean weight gain was in accordance with the recommendation of the Institute of Medicine (34) according to the pre-gestational BMI.

Our hypothesis was that the increased postpartum PRL levels in the lactation group mediated, at least partially, the improvement in MIR, as suggested by previous studies (4, 16). *In vitro* studies have suggested that pancreatic β cells have PRL receptors that stimulate cell proliferation and that lower the threshold of glucose-stimulated insulin release (15, 34–36). The final effect of PRL on insulin secretion/sensitivity, however, is believed to depend on its concentration: while higher levels of PRL within physiological concentrations (such as what occurs in pregnancy and puerperium) are associated with improved insulin sensitivity and glucose metabolism and lower prevalence of MS (37, 38), severe hyperprolactinemia (seen in pathological states such as prolactinomas) is associated with insulin resistance due to downregulation of insulin receptors (39). In nonpregnant individuals, populational studies also report an inverse association between PRL concentrations and prevalent T2DM (37, 40), even though the epidemiological data alone are not enough to prove causality. PRL levels<100 ng/ml are suggested to be, by some authors, the physiological levels in the early postpartum period (41, 42).

The concept of dose-dependent effect of PRL was demonstrated in a cohort study by Zhang et al. (4) in which postpartum PRL levels were divided into two groups:<100 ng/ml and ≥100 ng/ml. Each group was then subdivided into quartiles. Higher quartiles of PRL in the<100 ng/ml group were associated with lower fasting blood glucose and 2-h post-blood glucose levels, whereas in the higher quartiles of PRL in the ≥100 ng/ml group, the same association was not seen. Metabolomic and proteomic analysis were also performed in these patients and a more favorable lipid profile was found in patients with higher PRL levels in the<100 ng/ml group, suggesting that PRL indeed plays a role in improved metabolic profile.

In our study, the lactation group had significantly higher PRL levels [47.8 (29.6–88.2) vs. 20.0 (12.2–33.8), *p*< 0.001], but within the physiological range purposed by other authors as described above. We theorized that these higher physiologic PRL levels in the lactation group would mediate, at least partially, the improvement in glucose metabolism and in MIR. In the non-lactation group, a “relative hypoprolactinemia” ensues and could help explain worst results in MIR that were found. This theory is in accordance with a populational study performed by Wang et al. in which the prevalence of T2DM and impaired glucose tolerance (IGT) of 2,377 men and women older than 40 years and without hyperprolactinemia was evaluated. Higher PRL levels were significantly associated with lower risk of prevalent T2DM (odds ratio of 0.54 [95% CI 0.33–0.89]) and IGT (odds ratio of 0.38 [95% CI 0.24–0.59]) in the population (37).

The mediation analysis from our study, however, did not corroborate data from previous studies, as PRL was not found to significantly interfere in the association between breastfeeding and glucose metabolism and MIR.

The mechanisms underlying the protective effects of breastfeeding on the mother are obviously complex and most probably multifactorial. Elevated PRL levels in lactating women are only one of the possible explanations for the improved metabolic profile associated with breastfeeding. Other mechanisms cannot be excluded. For instance, oxytocin, a hormone produced during lactation, decreases food intake and has direct effects on adipocytes, inducing lipolysis, increasing brown adipose tissue adipogenesis and reducing visceral and liver fat deposition (43, 44). Besides that, it also increases glucose uptake in peripheral organs such as skeletal muscle (45) and stimulates insulin secretion. Taken together, these actions of oxytocin can potentially improve glucose metabolism and insulin resistance in lactating women. These aspects were not the objective of the actual study, but deserve attention in future studies and analysis.

Our study has limitations and strengths. The number of participants was small and only one blood sample was analyzed. Besides that, women were breastfeeding for a relatively short time (60 to 180 days); thus, it is possible that if women were breastfeeding for a longer period, different results could have been found. Our evaluation of breastfeeding was subjective, as there were no formal criteria for evaluating intensity and duration of lactation (since babies were young, we considered that they were breastfeeding on demand). We also did not specify the last time each woman had breastfed before the blood withdrawal, which could potentially interfere in the PRL values that were found. Another limitation of our study is not having other variables that represent possible mechanisms for the benefits of breastfeeding in metabolism, such as oxytocin or activation of brown adipose tissue, highlighting the importance of studies in this area that could evaluate these possible mechanisms along the same sample.

Even though our population size was small, groups were very homogeneous in several aspects that could have otherwise confounded the results, such as daily caloric intake, physical activity, and weight loss at postpartum. This homogeneity strengthens our hypothesis that lactation *per se* influences the improvement in glucose levels and MIR. A strength of our study that needs to be highlighted is its methodological approach. Use of DAGs in causal inference field is growing in the literature and has been considered a robust analytical strategy. The use of DAGs aims to decide which confounders should be left in the final model, thus trying to avoid bias by over-confounding or over-adjustments. All the potential known confounders were included in the DAG model (**Figure S1**), and the recommended variables were considered for the adjustments in the final model (**Table 3**). Another strength of this study is that we used mediation analysis to pursue the hypothesis of the role of PRL in this association. The mediation analysis considers the direct and indirect effects, the percentage of mediation, and the potential confounders of each step (in the direct and indirect association). This statistical information is not available when performing a linear regression analysis adjusted for a potential mediator. In this sense, the mediation analysis assesses a possible mediation more robustly and rigorously.

This is, to the best of our knowledge, the first study to evaluate the mediation effect of PRL on improvement in glucose metabolism and MIR in breastfeeding women with and without gestational diabetes. We believe that other studies are necessary to better elucidate the potential effect of PRL on MIR.

In conclusion, we observed that exclusive or predominant breastfeeding is associated with an improvement in glucose metabolism and MIR as soon as 60 to 180 days postpartum in women with a high risk for glucose intolerance, such as those with overweight, obesity, and/or GDM, even when adjusted for several confounders. PRL levels were not found to mediate the association between breastfeeding and improvement in MIR.

## Data availability statement

The raw data supporting the conclusions of this article will be made available by the authors, without undue reservation.

## Ethics statement

The institutional ethics committee of the Federal University of São Paulo approved the study (Protocol Number: CAAE: 89108618.0.0000.5505). The patients/participants provided their written informed consent to participate in this study.

## Author contributions

JO: first author, collection and interpretation of data, statistical analysis, and manuscript writing. PD: work design, collection of data, and manuscript revision. AF: statistical analysis, interpretation of data, and manuscript revision. CC: collection of data and manuscript revision. RM: collection of data and manuscript revision. SD: interpretation of data and manuscript revision. BA: senior author, work design, interpretation of data, statistical analysis, and manuscript revision. All authors contributed to the article and approved the submitted version.
