# Supplementary figures and images for: Prolactin does not seem to mediate the improvement on insulin resistance markers and blood glucose levels related to breastfeeding
Source: Front Endocrinol (Lausanne). 2023 Aug 30;14:1219119. doi: 10.3389/fendo.2023.1219119 (PMC10499379; doi:10.3389/fendo.2023.1219119)

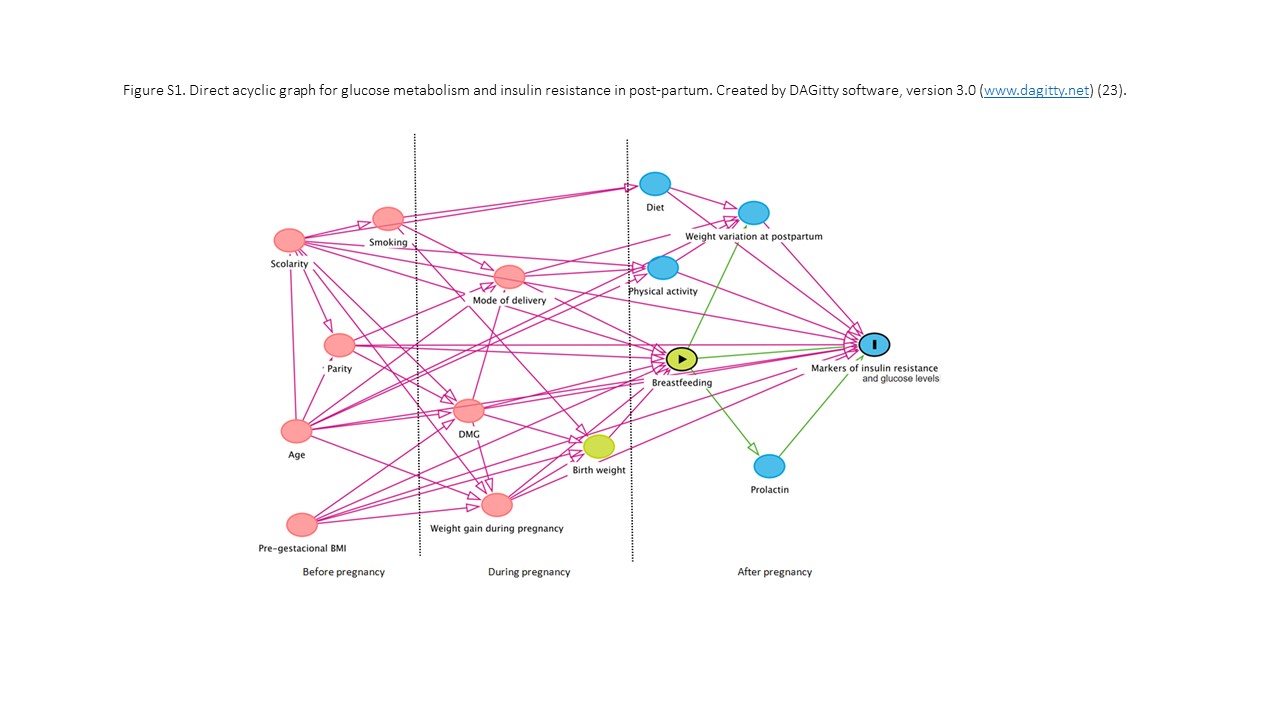

Supplement: Supplementary file 1 [file Image_1.jpeg]
