# Supplementary material for: Prolactin does not seem to mediate the improvement on insulin resistance markers and blood glucose levels related to breastfeeding
Source: Front Endocrinol (Lausanne). 2023 Aug 30;14:1219119. doi: 10.3389/fendo.2023.1219119 (PMC10499379; doi:10.3389/fendo.2023.1219119)
Supplement: Supplementary file 2 [file Table_1.doc]

SUPPLEMENTAL MATERIAL

Table 1S. Correlation of prolactin levels with fasting glucose and MIR (fasting insulin, HOMA-IR, TyG index and TG/HDL ratio).

|  | **Pearson coefficient** | **P** |
| --- | --- | --- |
| **Fasting glucose** | -0.139 | 0.181 |
| **Fasting Insulin**^#^ | -0.164 | 0.117 |
| **HOMA-IR**^#^ | -0.177 | 0.091 |
| **TyG index**^#^ | -0.226 | 0.028 |
| **TG/HDL ratio**^#^ | -0.243 | 0.018 |

**^#^**Log-transformed values of outcomes for analyses. Fasting glucose in mg/dL.
